# Psychosocial experiences of mothers caring for children with cerebral palsy in the eThekwini district

**DOI:** 10.4102/hsag.v28i0.2072

**Published:** 2023-05-22

**Authors:** Sibongile Seroke, Sipho W. Mkhize

**Affiliations:** 1 Department of Nursing, School of Nursing and Public Health, University of KwaZulu-Natal, Durban, South Africa

**Keywords:** cerebral palsy, challenges, disability, experiences, mothers

## Abstract

**Background:**

Cerebral palsy (CP) is the most prevalent neurological illness in children, and it can cause permanent sensory, motor and cognitive problems for the rest of one’s life. Raising a child with special needs necessitates extensive resources. Women in the middle and lower income brackets are more likely to care for children with CP.

**Aim:**

To explore and describe the psychosocial experiences of mothers of children with CP in eThekwini.

**Setting:**

This study was conducted at KwaZulu-Natal Children’s Hospital and rehabilitation centre.

**Methods:**

The research methods were exploratory and descriptive in nature, with a qualitative approach. Purposive convenience sampling was used to select 12 participants who were parents of children with CP under the age of 18. For data collection, semistructured interviews were utilised. The purpose of thematic analysis is to uncover, analyse and summarise themes and patterns within a data set. Semistructured interviews were used to collect data.

**Results:**

The psychosocial experiences of mothers of children with CP revealed three key themes. Themes included the burden of care, a lack of social support and the impact of children with CP on mothers.

**Conclusion:**

Participants whose children with CP experienced physical, emotional, psychological and social issues, including inaccessible services and buildings and social isolation from family, friends and the community.

**Contribution:**

This study helps to strengthen the development and review of policies on care, support interventions and mother empowerment for children with CP.

## Introduction

Cerebral palsy (CP) is a nonprogressive disorder of posture or movement caused by a lesion of the developing brain (Bulekbayeva et al. 2017; Oskoui et al. 2013). Children with CP have a higher mortality rate and numerous comorbid conditions (Strauss 2010). Several causes of CP have been identified in the literature, including teratogenic exposure during pregnancy, traumatic birth injury resulting in severe asphyxia, premature delivery, diabetes mellitus and radiation exposure (Burton 2015; Stavsky et al. 2017). Cerebral palsy has been found to be even more prevalent in Africa, with an estimated prevalence of 2–10 cases per 1 000 births (Donald et al. 2014:30). There is evidence that it is becoming a major issue for the continent (Pretorius & Steadman 2018). A high prevalence of CP was found in a Ugandan study (Kakooza-Mwesige 2017). Unfortunately, large knowledge gaps on aetiology, rehabilitation and treatment still exist in most African countries because of the prevailing cultural expectations and norms. Cerebral palsy is understudied on the African continent, and many countries lack registries (Burton 2015; Donald et al. 2014). The main feature of CP is impaired motor function, which affects children’s mobility (Braun et al. 2016; Sellier et al. 2015). Children with CP are more likely than other types of disabled children to develop long-term disabilities such as sensory, motor and cognitive deficits (Braun et al. 2015; Centers for Disease Control and Prevention 2012). Children with CP are primarily cared for by their mothers. Taking care of a family while also caring for a child with CP is common for these mothers, especially in lower and middle-income families, and the influence of gender stereotypes, poverty, societal shame and caring for a child with disabilities places significant strain on mothers caring for children with CP (Aisen et al. 2011).

It can be difficult to provide the high levels of care that a child requires when he or she has long-term functional limitations. Primary caregivers have reported that children with CP require a high level of physical and emotional care. A study from Ghana by Olawale, Deih and Yaadar (2013) suggests that primary caregivers may require education, training and exposure to support services available to improve the well-being of children with CP. Parents of children with CP in low-income countries have limited information about rehabilitation, training and feeding of their children (Polack et al. 2018; Zuurmond et al. 2019). Parents of children with CP usually experience higher stress, anxiety and depressive symptoms compared with parents of typically developing children (Davis et al. 2021; Parkes et al. 2011). Both parents are affected equally by CP, but mothers bear a disproportionate share of the burden because of their role as the child’s primary caregiver. Their caregiving duties hinder them from focusing on their careers or even actively seeking employment. A lack of formal and informal support systems compounded the challenges experienced by caregivers of children with CP. In the absence of comprehensive resources, families caring for children with CP must rely on the informal, interpersonal assistance of their social networks (Zuurmond et al. 2018). Several studies have found that parents and families with children with CP come from a wide range of socio-economic backgrounds. Singogo, Mweshi and Rhoda (2015) demonstrated that the majority of research on the challenges faced by caregivers of children with CP has been conducted in high-income countries.

The majority of disabled children live particularly in rural areas (Neille & Penn 2015). The caregivers of CP children in low- and middle-income countries may face unique challenges, so understanding their perspectives is critical. According to Wegner and Rhoda’s (2015) study, some African communities have negative attitudes and preconceptions about people with disabilities, believing they are God’s punishment. Because the majority of current research is being conducted in high-income countries, little is known about the difficulties that low-income women face when caring for a child with CP. The researchers carried out this study in the eThekwini District to collect first-hand accounts from mothers about what it is like to care for a child with CP.

## Aim

The aim of the study was to explore the psychosocial challenges experienced by mothers who care for children with CP in the eThekwini District of KwaZulu-Natal province.

## Methods

The research methods used were exploratory and descriptive in nature, with a qualitative approach. Purposive convenience sampling was used to select 12 participants whose children with CP were under the age of 18. Semistructured interviews were used to collect data. Data collection took place at the rehabilitation centre of KwaZulu-Natal Children’s Hospital.

The research was exploratory and descriptive, with a qualitative approach. The researcher chose this design to capture as much rich personal information as possible in accordance with the objectives of the study. Mothers of children with CP used group sessions to share their own experiences. All participants were told that their participation in the study was voluntary and that their decision to leave at any time would not affect their future treatment.

### Data collection

Four focus group sessions were conducted with 12 mothers of children with CP in a neutral place, structured to ensure confidentiality. Participation was completely voluntary. Predetermined short, open-ended and nonthreatening questions were used to elicit responses that reflected participants’ beliefs as well as experiences of raising children with CP. Each focus group lasted between 45 min and 90 min; the sample size was decided by the data saturation throughout the interviews. The researcher invited participants of all races to attend the focus group session, but only mothers who spoke isiZulu agreed to participate in the study, which was conducted in isiZulu and afterwards translated into English (Table 1). Focus group discussions were used as a qualitative approach to gain an in-depth understanding of social issues. The method aimed to obtain data from a purposely selected group of individuals rather than from a statistically representative sample of a broader population (Nyumba et al. 2018; Rosenthal 2016).

**TABLE 1:** Biography of the 12 participants.

Pseudonym	Age	Marital status	Child’s age	Source of income
FGP1	33	Single	10	Disability grant
FGP2	23	Single	3	Disability grant
FGP3	41	Single	6	Disability grant
FGP4	29	Single	3	Disability grant
FGP5	33	Single	7	Disability grant
FGP6	27	Single	2	Disability grant
FGP7	35	Single	9	Disability grant
FGP8	46	Married	10	Disability grant
FGP9	24	Single	4	Disability grant
FGP10	26	Single	8	Disability grant
FGP11	48	Widow	2	Disability grant
FGP12	38	Single	5	Father

### Trustworthiness

In this study, the process of credibility, dependability, conformability and transferability were part of the study population used to establish trustworthiness (Birt et al. 2016; Harvey 2015; Morse 2015; Nowell et al. 2017). The researcher guaranteed credibility by peer debriefing, member verification of acquired data and providing a detailed explanation of the environment (Braun et al. 2015; Morrow 2004). Familiarity developed by involving participants who were willing to genuinely take part in the interview, and probes were used during the interviews to ensure honesty from participants (Julien & Pecoskie 2000). *Dependability* evaluated the possibility that the same outcomes would be attained if the research were repeated using the same population, techniques and environment. This was achieved by clearly outlining the processes used in the study during data collection and thorough evaluation of the effectiveness of the enquiry (Babbie & Mouton 2015). Regarding *conformability*, according to Babbie and Mouton (2015:278), appropriate information about the sources should be left to establish if the findings and interpretations can be traced. The researcher analysed the raw data in the voice recorder, which was then translated by two independent language specialists from isiZulu to English and back to isiZulu to validate that the data were conveying the same meaning. The researcher read and reread the translated transcript to become acquainted with and comprehend the data’s substance. The researcher began the process of data analysis by coding the quotes from participants and grouping the codes into the pre-existing themes and emerging themes (Javadi & Zarea 2016). Findings, conclusions and a final report were written up to see how much the results came from the focus of the inquiry and not from the researcher’s own biases (Babbie & Mouton 2015:277; Bless et al. 2013:236).

*Transferability* is the degree to which the research’s results may be extended to other situations or with different respondents (Babbie & Mouton 2015:227). This was accomplished by describing the data with sufficient clarity and accuracy to allow other readers to evaluate the findings and explore their relevance in different circumstances. In addition, *purposeful convenient* sampling was utilised, which maximises the breadth of information by including women facing a variety of obstacles. By doing so, the study’s results may be applied in different situations or to other respondents in comparable positions (Patton 2002).

### Data analysis

**FIGURE 1:**
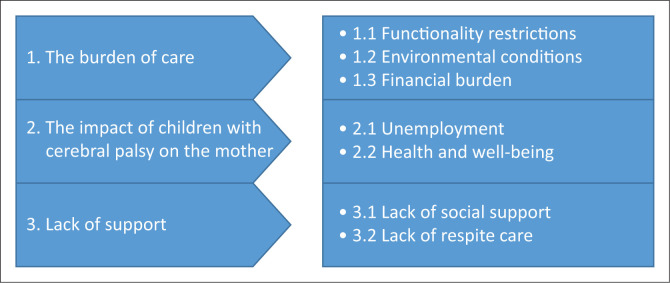
The main themes and sub-themes.

The researcher and the transcriptionist were the only individuals who had access to the voice recordings. While carefully listening to the interview tape, the researchers transcribed the data. The original interviews took place in isiZulu, the participants’ indigenous language, and they were translated and transcribed into English. The researchers performed a content analysis on a theme level (see emerging themes in Figure 1). The researcher examined the interview transcripts and coded the various remarks in the transcripts using open coding. This article’s text was formatted using a Microsoft Excel spreadsheet (Microsoft Corporation, Redmond, Washington, United States) (Bree & Gallagher 2016). Codes were initially sorted into key subjects following the initial phase of coding. Thematic analysis is a technique for identifying, analysing and reporting on patterns or themes in a data set. This type of analysis applies to any endeavour that tries to gain knowledge via the interpretation of data following Braun and Clarke’s (2014) six-step technique, which involves getting acquainted with the data, developing initial codes, using codes to create themes, evaluating the themes, identifying and defining the themes and generating a final report based on these findings.

### Ethical considerations

Ethical clearance to conduct this study was obtained from the Biomedical Research Ethics Committee of the University of KwaZulu-Natal (ref. no. REC-290408-009) and gatekeeping from the healthcare establishment granted accreditation to conduct the study. All participants were assured of confidentiality, and the material was transcribed using pseudonyms rather than the participants’ true names, for example, Focus Group Participant 1 (FGP1–FGP12). Participants were advised of their right to anonymity, confidentiality and withdrawal from the research at any time.

### Theme 1: The burden of care

During the focus group discussion interviews, the most often mentioned issue was the burden of caring for children with CP. This theme’s concerns included the child’s functional limitations, unmet demands and the cost of caring. The burden of care is divided into several sub-themes, each of which is discussed in depth below. The text in square brackets are the English translations of the quotes for clarity in reading.

#### Sub-theme 1.1: Functionality restrictions

Because of the functionality restrictions, the child is unable to perform daily tasks on his or her own. A mother describes how she feels in this situation in the following comment:

*‘Ingane iyagezwa, iyafunzwa, ayikwazi ukuzihlalele, iyashintshwa inabukeni konke lokhu kwenziwa imina.’* [The child cannot feed, bath, and change diapers this is my responsibility.] (FGP2, age 23, single [author’s own translation])

#### Sub-theme 1.2: Environmental conditions

The distance between home and the rehabilitation centre, as well as road conditions, medical supplies and assistive technology available in the area, were among the sub-themes identified as having a negative impact on the mothers. The following is how one person expressed her thoughts on the state of roads and transportation:

*‘Izimoto azingeni lapha ngihlala khona ngenxa yesimo esibi somgwaqo.’* [Transportation services are unable to reach my home due to poor road conditions in my neighbourhood; they claim the route is dangerous for their vehicle.] (FGP4, age 29, single [author’s own translation])

A physiotherapist gave a buggy to five mothers to help correct their children’s posture, but they found it difficult to store because their homes are informal, small house or Reconstruction and Development Programme (RDP) homes:

*‘Ingane yanikwa isihlalo iPhysiotherapist ukuze ihlale kahle iqonde kodwa inezinqinamba ngoba asikwazi ukusivala sisivule futhi nendawo yokusibeka endlini yeRDP incane.’* [I have a problem because my house is small, informal, or RDP, and there is no space for storage of the supportive device.] (FGP10, age 26, single [author’s own translation])

*‘Ngifaka umntwana kwi buggy ngoba umzimba namthambo akhe aqinile angikwazi ukumbeletha ngimphushe ngimhambisa emtholampilo noma kukude ngimele ngimhambise.’* [I will have to strap the child into the stroller and push her myself unless I can find someone to drive us. While the distance is great and you cannot walk there, we have done it several times.] (FGP2, age 23, single [author’s own translation])

#### Sub-theme 1.3: Financial burden

Many participants mentioned a lack of funds for necessities. Ten of the 12 mothers in this study admitted to financial difficulties, despite receiving a care dependency grant from their state to assist them with their caring responsibilities. The following are some of the mother’s comments:

*‘Ingane yami idla nge tube ngoba ayikwazi ukugwinya nokuhlafuna kufanele ngithenge ukudla okuhlukile, kuyabiza ngoba ngingedwa.’* [Because my child is unable to eat or drink, I must purchase a variety of special dietary supplements that can only be delivered through a tube in his stomach, which can be quite costly for a single mother.](FGP11, age 48, widow [author’s own translation])

*‘Ingane ihamba esibhedlela ukululwa amathambo kumele ngiye kanye ngesonto ngiphinde kuSpeech therapy ngolunye usuku esontweni elilodwa, ngiba nenkinga yemali yokuqasha imoto noma yomuntu uzongiphelezela ngoba angisebenzi nesibonelelo sikahulumeni sinakekela yonke umndeni.’* [The child must attend at least one weekly physiotherapy session and one weekly speech therapy session according to the therapist because I don’t work and my family relies on social security benefits, having to hire a vehicle or pay someone else to accompany me because I can’t carry my child on my back is a financial burden.] (FGP6, age 27, single [author’s own translation])

One participant described her situation as a result of her partner’s lack of financial assistance and the fact that he had rejected their child. Her clothes and food are provided by her family and friends.

*‘Ubaba wengane wangishiya ngimthola usizolokudla nezingubo ezihlobeni.’* [My boyfriend abandoned the child; my family help me with clothes and food.] (FGP10, age 26, single [author’s own translation])

### Theme 2: The impact of children with cerebral palsy on the mother

The main consequences of having a child with CP that came up during the interview were employment difficulties, the mother’s poor health and isolation.

#### Sub-theme 2.1: Unemployment

Most of the participants in this study had difficulty finding work and described the difficulties they faced while looking for work or working. Their children’s limitations varied, and they needed child minders to care for them while they were gone. The responses were as follows:

*‘Ngiyasaba ukushiya ingane nomuntu uzoyibheka usuku lonke ngenxa yesihluku engisibona kutelevision izingane zihlukunyezwa futhi angithembi ukuthi kukhona ongayibheka ngoba ikhubazekile.’* [I am afraid to leave my child with a child minder because I have seen some caregiver’s abuse children with disabilities on television. I don’t trust anyone else to look after their children.] (FGP9, age 24, single [author’s own translation])

*‘Angisebenzi ngihlala ekhaye ngibheke umfana wami. Kunezindleko eziningi ukubheka ingane ine CP. Ngesimo sokungabi nemali angisakwazi ukumuyisa ukuyolulwa amathambo, uyangaleso sikhathi uma ikhona.’* [Right now, I am not working. I am a stay-at-home parent for my child’s sake. It is prohibitively expensive to care for a child with this condition. Because of the financial crisis, I cannot even afford to take this child to physiotherapy, so I only do it occasionally.] (FGP11, age 48, widow [author’s own translation])

#### Sub-theme 2.2: Health and well-being

Five participants described what they went through after learning their children had CP. Raising a child with CP has been linked to both emotional and physical distress.

#### Sub-theme 2.2.1: Emotional distress

The descriptions of participants’ feelings following diagnosis revealed emotional pain. The following are some of the comments made by participants.

*‘Ingane yami yahlinzwa kulungiswa inhliziyo ngatshelwa ukuthi ine VSD yayiphila kahle yaphuma ikhala ngiyibeletha. Emva kokuhlinzwa ingane ayisenzi lutho ayisanceli, idla nge tube. Ngahlala esibhedlela isikhathi eside. Dokotela wangitshela ukuthi isikhubazekile ngenxa yokusweleka kweoxygen. Ngeke isakhula njengezinye izingane ngababuhlungu kakhulu ngeke ngalukhohlwa lolo suku. Ngayiswa esibhedlela ukuyolulekwa.’* [My child was born normally, cried at birth, and I was later informed that he has a heart problem that will require VSD [ventricular septal defect] repair. For a few days after the operation, he was unresponsive, not feeding or crying. When the doctor told me that my child’s brain had not received enough oxygen and that his growth and development would be delayed, I was shocked and enraged. I had no idea I’d have a disabled child. I was referred to a therapist because I was emotionally disturbed.] (FGP11, age 48, widow [author’s own translation])

#### Sub-theme 2.2.2: Physical health

Some participants reported body aches as a result of assisting disabled children; they believe that the lack of assistance devices, such as a buggy or wheelchair, contributes to their body pains as they are required to carry the children.

*‘Ingane ayikwazi ukuhamba kubele ngiyithwale uma iya endlini encane, ngiyayigeza konke iyenzelwa. Ngihamba ibanga elide ngezinyawo ngiyibelethile uma ngiyisa kwimobile clinic, ngibambe ulayini futhi azikho nezihlalo, kuba buhlungu umzimba wonke.’* [Because my child cannot walk, I must constantly lift her to use the rest room, bathe her, and do anything else. I walk a long distance on foot, carrying him on my back, to and from the mobile clinic. I need to get in line, but there are no chairs available. I am plagued by aches and pains all the time.] (FGP7, age 35, single [author’s own translation])

### Theme 3: Lack of support

A lack of support was identified as a problem, as the question of whether they receive support from family, community members and hospital staff was raised.

#### Sub-theme 3.1: Lack of social support

Ten mothers mentioned a lack of social support, as evidenced by the following quotes:

*‘Ngiyafisa sengathi ngingaba nomuntu ongangisiza ukunakekela umntwana, ngimnakekela ngedwa Uma ngimthola usizo kumuntu uye afune ukukhokhelwa.’* [I wish I had someone who could also assist me. I have to do more now because I have a cerebral palsy child and no one can help me; if they come and help, they expect to be compensated.’] (FGP11, age 48, widow [author’s own translation])

One participant also mentioned how having a child with CP made them feel alone.

*‘Abangane bayafika bangivakashele ekhaya badlalise ingane basheshe bahambe, kodwa angisenabo abangane abaningi.’* [Friends come to visit and play with the child, but I do not have many friends right now, she says.] (FGP2, age 23, single [author’s own translation])

*‘Angisakwazi ukuya ndawo ngoba nginomntwana okhubazedkile, uma ngifisa ukuvakashe kumele ngicabange ukuthi ngizomshiya nobani ngoba ungumthwalo wami.’* [I am unable to travel because I have a disabled child. First, I must remember that if I am to go anywhere, he is my responsibility.] (FGP11, age 48, widow [author’s own translation])

#### Sub-theme 3.2: Lack of respite care

All participants mentioned respite care centres that could provide comprehensive care in the form of intensive rehabilitation and respite care. This is what came up during the discussion, as stated below:

*‘Kumele kube nesikhungo esizonakekela abantwana nathi singabazali sikwazi ukubakhona. Sizofunda ukubelula amathambo, kufanele belulwe kanye ngosuku kodwa isibhedlela sibabona kanye ngenyanga, ngesinye isikhathi asibahambisi ngezizathu ezahlukene.’* [I believe there should be a rehabilitation centre to care for our children, and we as parents should be allowed to be with them so we can learn how to do exercises. They need to do daily physiotherapy at the hospital physiotherapy is done once a month, which sometimes I fail to go due to various reasons.] (FGP3, age 41, single [author’s own translation])

## Discussion of the findings

It has been stated that the health and well-being of primary caregivers often has an influence on the well-being of the individuals with disabilities in their care (Brehaut et al. 2004; Raina et al. 2005). The majority of studies that have explored the experiences of caregivers for individuals with CP have been conducted in developed countries, including Australia, Ireland and Spain (Byrne et al. 2010; Davis et al. 2009; Fernández-Alcántara et al. 2014; Whittingham et al. 2011), while fewer studies have explored the caregiver experience in developing countries, including Kenya, South Africa, Thailand and Uganda (Barratt & Penn 2009; Geere et al. 2012; Gona et al. 2010; Hartley et al. 2005; Huang et al. 2011; Ngubane & Chetty 2017). The current research sought to evaluate the obstacles faced by mothers caring for children with CP in the eThekwini District, KwaZulu-Natal.

Studies have shown that parents who take care of children with disabilities often face a number of problems, but they also find a number of resources that help them do their jobs (Bourke-Taylor et al. 2010; Davis et al. 2009; Golden & Nageswaran 2012; Green 2007; McManus et al. 2006; Murphy et al. 2006; Myers et al. 2009; Resch et al. 2010; Whittingham et al. 2011; Yantzi et al. 2006). In this study, too, it was found that mothers who took part had to deal with a number of problems. The following themes and sub-themes emerged from this study.

### Increased burden of care

Mothers in this study discussed their experiences and challenges combining the daily care of their children with CP, their siblings and other family responsibilities. Most of them shared having no time of their own, for their other children and for their partners or spouse. Mothers in this study stated how their children’s functional limitations made it necessary for them to receive help with activities of daily living, such as eating, bathing, and motions associated with walking. The increased burden of care may lead to more psychological challenges than with children with chronic illnesses, as identified in the literature of Viljesh and Sukumaran (2007:79) and Cheshire, Barlow and Powell (2010:1673). Bumin, Günal and Tükel (2008:192), Garip et al. (2017) and Ones et al. (2005:250) concurred with the findings of the study, which stated that high levels of anxiety are caused by the burden of being a caregiver, chronic fatigue, negative social attitudes, social environmental issues and a lack of implementation of policies on childhood disabilities. In their research, Al-Gamal and Long (2013:624) revealed that caregivers of disabled children are more likely to suffer from depression, emotional discomfort, cognitive impairments and poor mental health than mothers of children without disabilities.

The caregiver’s mental health is negatively impacted by the prevalence of children’s disabilities, behavioural issues, bad temperament and deficiencies in cognitive and sensory functions (Davis 2010:70, 2012:557). Marrón (2012:769) considered depression to be an important indicator of burden of care and quality of life. This view was supported by the results of a Sri Lankan study, which revealed psychological disorders, namely depression and anxiety, in cases where the burden of caring for children with CP was high (Vadivelan et al. 2020; Wijesinghe, Hewage & Fonseka 2014:10). One would presume that parents of children with milder forms of CP would be less stressed compared with those with children who have more severe disabilities. However, studies in various well-resourced and under-resourced countries failed to show any relationship between severity of the child’s physical disability and caregiver stress levels, psychological well-being and quality of life (Brehaut et al. 2009:184; Hamzat & Mordi 2007:192; Ones et al. 2005:234; Purgin 2007:6; Sawyer et al. 2011:341; Skok, Harvey & Reddihough 2006:55). In a number of instances, it has been found that mothers display mixed feelings towards a diagnosis of CP, where they would either be relieved to find the cause for their child’s condition or grieve the loss of the healthy child that they were expecting to have (Huang, Kellet & St John 2010; Whittingham et al. 2013:1215). One of the mothers in this research suffered from despair and emotional anguish since her child was born healthy but developed CP after undergoing surgery. These parents and guardians struggled to accept the fact that their child, who seemed to be healthy at birth, would not reach the same developmental milestones as their peers (Fernández Alcántara et al. 2014; Masood et al. 2015).

### The unmet needs

Needs that have been mentioned by mothers in this study refer to difficulties in accessing services and insufficient supply of physical aids. The results indicated that it was difficult for children with CP to gain access to the necessary services and adapted equipment. Numerous studies have shown that a significant proportion of the demands of children with CP are unmet, hence increasing the hardship suffered by their mothers (Diab Eloreidi et al. 2021; Uldall 2013:203).

The inability to access assistance has exacerbated the problems of caring for children with CP and increased caregiver stress, making it harder to manage. The challenges to access services timely and frequently were reported as on of the unmet needs. Almost a majority of the women relied on public transportation to go to appointments or take their children on trips; only one participant’s spouse could help with transportation. They would have to walk to the clinic and push their children in their wheelchair, if the child had one. A few mothers complained of musculoskeletal challenges, such as backaches and general body pains, because they had to put their children on their backs or carry them, and the children were often too heavy to be carried. Even if they hired a taxi, they had no proper driveway into their homes; they had to put children on their backs to be able to get to the taxi or the bus. For the children who had a buggy or wheelchair, their houses were too small to keep them. The majority of the participants were from peri-urban areas, residing in RDP houses; a few were renting and others shared two rooms with their families. These findings support the studies by Singogo et al. (2015:5) and Badu et al. (2016), who discovered that nearly all mothers experienced physical access challenges owing to the natural geography, architectural features in the built environment and transport challenges; these included narrow or no sidewalks, narrow doorways, an absence of ramps, no or broken lifts and small indoor spaces. Some of the participants identified the need for assistive devices or wheelchairs, and some identified the need to be provided with diapers. Some of the physical challenges mothers reported were a lack of proper housing, since most of them lived in small RDP houses; the structures were not friendly, especially to those who used wheelchairs. The houses were also small and they had big families, thereby experiencing congestion. It is therefore important that the department responsible for road maintenance ensure that the access roads within these residential areas be upgraded to accommodate wheelchair and buggy users.

### Physical and emotional challenges

According to Hamzat and Mordi (2007:19320) and Dambi (2015:699), mothers experienced physical health problems related to stress, such as back pain and ulcers, which were prevalent in caregivers of children with CP. Emotional pain and depression were experienced by the majority of mothers as a result of having given birth to a child with CP and not clearly understanding the prognosis. One mother expressed that she lives in fear that the child might die, and another was worried about the delay in growth and development of her child, having the false hope that her child would reach normal milestones. Long-term caregiving has been proven to predispose carers to chronic strain, stress, anxiety, depression and discomfort compared with the rest of the population, as shown by both the literature and the answers of the participants (Brehaut et al. 2004, 2009; Cheshire, Barlow & Powell 2010; Davis et al. 2009; Navaie-Waliser et al. 2002; Sawyer et al. 2011).

### Lack of support

Mothers also cited the lack of support as a difficult aspect of caring for a child with CP. A few of the mothers had strong social support from families and friends, as mentioned during interviews, with whom they could leave the child with when they had to go to town, but a majority did not have such support. Family and friends did not know how to care for a child with disabilities, and the mother did not trust anybody to look after her child. Mothers described emotions of isolation and a lack of socialisation, which were exacerbated by the child’s mobility issues. The results of this study showed that it was hard for the mother to leave the house with her child because it took a great deal of planning, physical work and energy, as described by Yantzi, Rosenberg and McKeever (2007:47). Several mothers in this study, however, noted that when their child was accepted and included by members of their community (in some cases they were invited to attend birthday parties and one mother was invited to a picnic with her son), they experienced greater support and were less likely to feel alone. Mothers of children with disabilities in both developed and developing countries, such as those in Asia and Africa, have talked about these kinds of things (Dambi & Jelsma 2014:8; Geere 2013:389; Sandy, Kgole & Mavundla 2013:348). Communities in Africa often have a negative perception of people with disabilities because they believe it is a punishment from God (Urimbenshi & Rhoda 2011:404) and of a spiritual nature (Wegner & Rhoda 2015:6). Participants reported they received a care dependency grant, but they all agreed it is not enough to meet the demands of caring of the child with CP, such as diapers, special food and transportation to go for medical reviews. Some mothers shared that the grant was also used for the entire household, as there was no other steady income. Caring for a child with a disability places a substantial burden on the caregiver who assumes that responsibility. Basic costs like food, transportation, clothing and healthcare needs are included in the cost. Other costs may include respite care, specialised adaptive equipment, changes to the environment and therapeutic services (Bryns 2013:503–504; Ross & Deverell 2004:192). A number of studies have shown that the majority of children with CP are raised by poor families with unstable finances, who reside in rural communities where there is little access to appropriate services (Martha 2010:53; Mobarack et al. 2003:601). The results of a study carried out in the rural communities of South Africa reflected that although children with CP qualify for government assistance in the form of care dependency grants, the money is usually the family’s only source of income and not all the money is spent on the child (Harvey 2011:4). The child’s state benefit does not cover the expense of caring for the individual with the disability (Bryns 2013:504). Although there are costs involved in looking after a child with CP, caregivers often have less time available to work for pay or acquire work or sustaining employment (Brehaut 2004:184; Davis 2010:67; Pfeifer 2014:365). According to literature reviewed, time constraints also interfered with employment opportunities for Kenyan caregivers, preventing them from providing for their families (Gona 2010:33). In 2003, the Board of Directors of the American Academy of Pediatrics appointed a task force with a view to guiding policy and making recommendations for promoting functional families. One area of investigation was financial stability in these families. In this study, mothers indicated that financial burden emerged primarily because of unemployment as a result of the amount of time they were required to spend with their child on a daily basis, which concurred with the study findings of Bourke-Taylor et al. (2010). Findings have shown that female-headed families were five times more likely to live in poverty than those of married couples (Schor 2003:1543). The situation is similar in Africa, where female African caregivers are often unemployed and live in poverty. In a study conducted by Purgin (2007:12), the amount of income correlated strongly with stress levels in caregivers, leading her to conclude that poverty was a major contributing factor to stress. This conclusion concurred with findings of other researchers in well-resourced and resource-constrained countries (Guyard et al. 2012:1596; Mobarak et al. 2000:429). Even when caregivers of children with CP shared similar socio-economic circumstances to matched controls, a Nigerian study found they had less disposable income and demonstrated a poorer quality of life than the control group (Fatudimu 2013:133). In terms of the larger community, African communities frequently have negative attitudes towards, and even reject, people with disabilities because of beliefs that disability is caused by evil spirits (Wegner & Rhoda 2015), is a punishment from God or is because of involvement in witchcraft (Singogo et al. 2015). Some of the caregivers in this study reportedly believed they were bewitched during their pregnancy, while others saw their disabled child as a form of punishment. These cultural beliefs lead to the stigmatisation of people with disabilities and their families by members of the community (Akintola 2008; Dogbe et al. 2019). Caregivers also reported feelings of embarrassment and shame as a result of others’ negative reactions (Whittingham et al. 2011). These negative reactions and rejections were often found to affect the child as well as his or her caregiver.

### Financial burden

Financial burden was identified as another prominent challenge associated with caring for a child with CP. Items such as special food, clothing, transport and diapers were the most expensive to provide, which agrees with the findings obtained by Davis et al. (2010) that the most basic items required for the care of a severely disabled child can be the most difficult to provide. The mothers stated that the financial strain arose mostly as a result of their inability to work because of the amount of time they were required to spend with their child on a daily basis (Bourke-Taylor et al. 2010). Since mothers were unable to obtain employment because of the demands associated with their caring duties, the compounding effect of their unemployment status and financial burden often left them unable to provide the basic necessities for their children (Heymann & Kidman 2008). The lack of access to respite care was identified as a prominent support-related challenge that mothers experienced, and financial burden was another prominent barrier that was associated with caring for a child with CP.

### Identified needs

Lack of access to respite care was identified as a prominent support-related barrier that caregivers experienced. Most mothers mentioned affordability, as the special schools that could take care of their children were only available in urban areas. There is a 2-year waiting period for admission, and even then they are not guaranteed a space. The literature highlighted the importance of respite services, as caregivers are often only able to take a break from their caring duties when their child attends a care facility or school for a few hours a day or a week (Ogunlana et al. 2019; Yantzi, Rosenberg & McKeever 2007:47). Although most caregivers found it challenging to access adequate respite services, there were some caregivers who were able to find an appropriate care facility for their child in their community. These caregivers recognised the importance of their child’s crèche, school or day-care, as they valued the time off from their caring duties that these facilities afforded them (Yantzi et al. 2007). Furthermore, caregivers also valued the contributions that staff at their child’s care facility were making to their child’s progress. A collaborative relationship was often developed between their child and the staff at the care facility, and staff members often shared valuable knowledge that allowed parents to understand the cause of their child’s disability as well as the therapies that could be utilised in order to manage it (Leiter 2004; Whitmore & Snethen 2018).

### Lack of social services

Mothers mentioned they did not understand the diagnosis of CP and received different information about the prognosis from different health practitioners. The lack of supportive counselling following the diagnosis was also identified. Social workers and rehabilitation teams should be available at primary health clinics to ensure continuity of care, support and well-being of the mother and the child. The attitude of health professionals, especially nurses, had been mentioned. Some nurses lacked skills on how to care for the child with CP, and reference was made to when the child had to be weighed and nurses doing the observations did not know how to weigh the child with CP using the normal scale. The mothers were concerned whether the child was gaining or losing weight. Some nurses referred to the mothers with their children’s diagnosis. All categories of nurses should be trained on how to care for a child with CP. Houses should be allocated to the family of a child with a disability by the local authority or by councillors, and they should be environmentally friendly and accessible, as requested by mothers, as this would alleviate stress and promote the well-being of the mothers. The application for care dependency grants should not become a lengthy process. and the assessment should not take too long to be completed. Social workers should assist with the processing of the application that would exempt mothers with children with CP from having to be assessed to qualify for social welfare programmes. Access to social welfare programmes may significantly contribute to the reduction of poverty for caregivers and promote adaptation to living with a child with CP.

Mothers suffer a burden of care because they must combine daily care for their children with CP, their siblings and other family responsibilities. The majority of them expressed a lack of time for themselves, their other children and their partners or marriages. Because of their functional limitations, the children required assistance with daily living activities such as bathing, feeding and motions associated with their capacity to walk; this was predominantly the mother’s job. According to the study’s findings, another important obstacle connected to caring for a child with CP was the financial load. Food, clothing, transportation and diapers were the most expensive to provide, consistent with Dambi, Jelsma and Mlambo’s (2015) findings was that the most basic commodities required for the care of a severely challenged child can be the most difficult to give. The financial load was mostly a result of their inability to find work because of the amount of time they were obliged to spend every day with their children (Dambi et al. 2015).

Physical access concerns were also found; almost all participants relied on buses and taxis to attend clinic or hospital appointments or take their children on trips; only one participant’s partner could assist with transportation. The residential apartments were inconveniently located, far from bus stops and taxi ranks. They would have to walk to the clinic while pushing or wheelchairing their children. Participants reported experiencing musculoskeletal difficulties, such as backaches and general body aches, as a result of carrying or putting their children on their backs, and the children were frequently too heavy to carry. Even if they hired a taxi, they lacked a decent driveway leading to their homes, forcing them to carry their children to the taxi or bus on their backs. The participant’s house has little space, and storage for a buggy or a wheelchair proved difficult. The overwhelming majority of participants were from peri-urban areas and lived in RDP houses; some rented, while others shared two-roomed dwellings with their families. Singogo et al. (2015) discovered that almost all the mothers faced physical access barriers as a result of natural geography, built environment and architectural features. Transportation obstacles include limited or nonexistent walkways, tiny doorways, a lack of ramps, no or malfunctioning elevators, and confined indoor spaces when the child taken from home to public places such clinics or supermarkets. As a result, the road maintenance sector is responsible for upgrading access routes within designated residential zones to accommodate wheelchair and buggy users.

## Conclusion

This study aimed to explore and identify psychosocial challenges experienced by mothers caring for children with CP in eThekwini District, KwaZulu-Natal. The study revealed that participants encountered a number of challenges that influenced their caring experiences. A number of challenges emerged, including the personal consequences of caregiving, difficulty adjusting to caregiving duties, environmental conditions, lack of access to healthcare services, lack of respite services and negative perceptions towards disability. Almost all themes that were identified were consistent with previous findings on caregiver experiences; it was found that there were a number of service-related challenges that were particularly experienced by participants. Firstly, the lack of information on the condition, prognosis and care of a child diagnosed with CP was a common barrier reported by participants. This challenge resulted in much confusion and often a mistrust of healthcare professionals. There is a need to ensure that mothers receive an explanation of the meaning of their child’s illness, how it was caused and how it may be controlled at a level that they can comprehend. Secondly, mothers struggled to adapt to their caring responsibilities and lacked suitable support networks. According to the results of this research, there is a need for programmes that offer women the support and information they need to manage their caregiving responsibilities. There is a need for home- or community-based interventions that might mother to be maintained throughout who are often unable to leave their homes owing to the demands of their care responsibilities. These sorts of programmes are not feasible because of a lack of finance and qualified personnel; thus, there is a need to enhance the supply of these services. Thirdly, participants identified a lack of disability-friendly services as an additional barrier to healthcare and respite services. Public transportation was pricey and ill-equipped to handle youngsters, according to mothers. This supports the idea that public transportation must be made more accessible by equipping cars with the essential supports for those using assistive devices. The provision of proper respite services is an essential resource for carers, as it would provide them with time away from their caring responsibilities, which often prevent them from engaging in social activities or seeking work.

## Recommendations

### Policymakers

The government should review the policies regarding financial support, disability allowances to address the child’s needs. The government should offer professional counselling services for carers with the goal of enhancing psychosocial care and promoting coping when stressful circumstances, depression and anxiety are experienced. There should be community-based rehabilitation programmes that have a respite care component. These programmes might eliminate the stigma associated with disability and raise awareness about it. Intervention care, such as family social support groups, should be introduced at rehabilitation centres for families with children who have CP, where they may meet other afflicted families and share stories, interact and trade ideas. The initiative might help families even more by providing short-term caregiving assistance.

### Nursing education

The midwifery curriculum must design a module that include comprehensive history taking and screening of all pregnant women using the basic antenatal care, which will assist with early detection of risk factors. Nursing training should cover how to render basic nursing care and procedures to children with disabilities. Nursing counselling skills should be strengthened in order to facilitate support groups at the primary and community healthcare centres. Learning outcomes should be incorporated in the prenatal care module that address risk factors that might predispose mothers to having a child with CP or other developmental impairments.

## Limitations of the study

Despite the fact that the sample was composed of mothers from diverse sections of eThekwini District with varying demographic features, the entire participant pool was not totally representative of the maternal population as a whole. This problem was reinforced by the selection of a small sample consisting of 12 mothers. All of the participating mothers resided in peri-urban settings; hence, the issues faced by mothers living in urban and rural areas were not investigated. The challenges faced by low-income and low-education parents may not be comparable to those faced by parents in the higher or medium-income brackets who are also well educated.

## Future study

Future studies could build on the findings of this study and consider them when developing interventions for caregivers of children with CP. They could consider the efficiency of services provided to children with disabilities and their families, to improve or strengthen these services for the betterment of quality of care for children with disabilities and their caregivers. The needs of families with children who have CP require identification and an understanding of the challenges they encounter accessing rehabilitation services and healthcare. Policies and the related concepts and effects of specific policy initiatives require further study.
